# Changes in liver stiffness values assessed using transient elastography in chronic hepatitis B patients treated with tenofovir disoproxil fumarate: a prospective observational study

**DOI:** 10.1186/s12876-023-02846-9

**Published:** 2023-06-15

**Authors:** Heejin Cho, Yun Bin Lee, Yeonjung Ha, Young Eun Chon, Mi Na Kim, Joo Ho Lee, Hana Park, Kyu Sung Rim, Seong Gyu Hwang

**Affiliations:** 1 Department of Internal Medicine and Liver Research Institute, Seoul National University Hospital, Seoul National University College of Medicine, 101 Daehak-ro, Jongno-gu, Seoul, 03080 Republic of Korea; 2 Department of Internal Medicine, CHA Bundang Medical Center, CHA University, Seongnam, Korea; 3 Health Screening and Promotion Center, Asan Medical Center, University of Ulsan College of Medicine, Seoul, Korea

**Keywords:** Liver stiffness, Transient elastography, Tenofovir disoproxil fumarate, Chronic hepatitis B

## Abstract

**Background/Aims:**

Regression of liver fibrosis during antiviral therapy in chronic hepatitis B (CHB) patients has been demonstrated, but data on the influence of long-term treatment with tenofovir disoproxil fumarate (TDF) on liver stiffness (LS) measured by transient elastography are scarce. We aimed to investigate the changes in LS values during the 144-week TDF therapy in treatment-naïve CHB patients.

**Methods:**

This prospective observational study was conducted from April 2015 to July 2020 at CHA Bundang Medical Center. Laboratory tests and LS measurements were performed at baseline and repeated at weeks 12, 24, 48, 96, and 144. A significant decline in LS was defined as ≥ 30% decrease in LS value at week 96 from baseline.

**Results:**

A total of 48 treatment-naïve CHB patients initiating TDF therapy were screened, and 36 patients were included in the final analysis (median age, 46 [interquartile range, 34.5–55.8] years; 19 men [52.8%]). During TDF therapy, the median LS values decreased from 13.8 kPa at baseline to 8.7 kPa, 6.5 kPa, and 6.4 kPa at weeks 48, 96, and 144, respectively (all *P* < 0.001). At week 96, virological and biochemical responses were achieved in 34 (94.4%) patients and 20 (76.9%) patients, respectively. Moreover, 21 of 36 (58.3%) patients showed a significant decline in LS value. A higher baseline LS value was a single independent predictor for the reduction in LS value at week 96 from baseline (*P* < 0.001).

**Conclusions:**

During the 144-week TDF therapy, LS values declined significantly in treatment-naïve CHB patients.

**Supplementary Information:**

The online version contains supplementary material available at 10.1186/s12876-023-02846-9.

## Introduction

Chronic hepatitis B virus (HBV) infection affects approximately 257 million individuals worldwide, of which only 27 million (10.5%) of people were diagnosed with chronic hepatitis B (CHB), and 4.5 million patients were treated [1]. Chronic HBV infection leads to morbidities and mortality with progression of liver fibrosis, hepatic decompensation, and development of hepatocellular carcinoma (HCC) [2–6]. Hepatic necroinflammation occurs secondary to the host’s immune response to the virus, which induces liver disease progression through the immune-active phase [7, 8]. Long-term antiviral treatment with potent nucleos(t)ide analogues (NAs) has been demonstrated to prevent the progression of liver disease by suppressing HBV replication and reducing chronic hepatic inflammation [9–13]. Several studies have reported the reduction of liver fibrosis proven by liver biopsy in CHB patients after long-term administration of NAs [10–13]. In a previous study evaluating the influence of long-term suppression of HBV replication on liver fibrosis, 348 CHB patients receiving antiviral therapy with tenofovir disoproxil fumarate (TDF) underwent liver biopsy at baseline and week 240 after initiation of antiviral therapy. The regression of fibrosis, defined as ≥ 1-point decrease in Ishak score, was shown in 176 of 348 (51%) patients, suggesting that long-term antiviral therapy with TDF may lead to fibrosis regression in CHB patients [10]. Therefore, monitoring the changes in the degree of liver fibrosis during antiviral treatment is important for evaluating the efficacy of antiviral treatment and the long-term prognosis of the liver disease.

Although liver biopsy is regarded as the gold standard for the assessment of the degree of liver fibrosis, liver biopsy is an invasive procedure that can cause various complications, such as pain, infection, and bleeding, thereby limiting its use in clinical practice [14–16]. Recently, transient elastography (TE), which is a simple, accurate, and non-invasive diagnostic method for evaluating liver fibrosis by measuring liver stiffness (LS), has been widely used in patients with chronic viral hepatitis. Previous studies showed that LS values significantly decreased during antiviral treatment with entecavir or lamivudine in CHB patients, suggesting improvement of liver fibrosis [17–19]. However, studies investigating the changes in LS values assessed by TE during long-term TDF therapy in patients with chronic HBV infection are scarce [20–22].

In this prospective observational study, we aimed to investigate whether liver fibrosis was improved during the 144-week antiviral treatment with TDF by measuring changes in LS values in patients with CHB.

## Materials and methods

### Patients

This prospective observational study was conducted at CHA Bundang Medical Center, Seongnam, Korea from April 2015 to July 2020. Inclusion criteria were as follows: (1) age between 20 and 75 years; (2) presence of the hepatitis B surface antigen (HBsAg) for more than 6 months (medical history of chronic HBV infection can be alternative); (3) clinically diagnosed liver cirrhosis, defined as a low platelet count below 100,000/mm^3^ accompanied by splenomegaly, presence of esophageal or gastric varices, or imaging findings suggesting liver cirrhosis, and HBV DNA level of > 2,000 IU/mL; hepatitis B e antigen (HBeAg)-positive non-cirrhotic patients who had HBV DNA level of > 20,000 IU/mL, an elevated alanine aminotransaminase (ALT) level of > 80 IU/L, and a baseline LS value of > 5.5 kPa; or HBeAg-negative non-cirrhotic patients who had HBV DNA level of > 2,000 IU/mL, an elevated ALT level of > 80 IU/L, and a baseline LS value of > 5.5 kPa. The exclusion criteria included (1) hepatic decompensation, defined as an elevated serum level of bilirubin of > 3 mg/dL, an international normalized ratio of > 1.6, a low serum albumin level of < 2.8 g/dL, history of ascites, variceal bleeding, or hepatic encephalopathy, or Child-Pugh score of ≥ 10; (2) prior antiviral treatment with interferon or other NAs; (3) decreased renal function with creatinine clearance of < 50 mL/min, which was estimated using the Cockcroft-Gault formula; (4) serious comorbidities, such as congestive heart failure, chronic kidney disease, hematologic disease, or malignancies including HCC; (5) coinfection with hepatitis C virus, hepatitis D virus or human immunodeficiency virus; (6) significant alcohol consumption (≥ 210 g per week in men and ≥ 140 g per week in women [23, 24]; (7) evidence of autoimmune hepatitis, hemochromatosis, or Wilson’s disease; (8) pregnant or breast-feeding women; and (9) prior organ transplantation including liver transplantation. The study protocol followed the ethical guidelines of the 1975 Declaration of Helsinki and was approved by the Institutional Review Boards of CHA Bundang Medical Center (IRB number: BD2014-178).

### LS measurement

Experienced technicians (> 20,000 examinations) measured LS values on the right lobe of the liver through the intercostal space after positioning patients in the dorsal decubitus position with the right arm in maximal abduction [25]. The LS values for each patient were obtained by performing 10 inspections using the 3.5 MHz standard M probe for patients with BMI < 25 kg/m^2^ and the 2.5 MHz XL probe for patients with BMI ≥ 25 kg/m^2^. The median values of at least 10 successful measurements of LS were used for the analysis and expressed as kilopascals (kPa). LS values were regarded as reliable when the interquartile range (IQR)/median value was less than 30%, and a success rate was more than 60% [26].

### Study procedures

Demographic data, such as age and sex, were collected and body mass index (BMI) was measured during screening. Clinical data, including aspartate aminotransferase (AST), ALT, gamma-glutamyl transferase (GGT), albumin, fasting glucose, international normalized ratio, platelet count, HBV DNA, HBeAg, and LS values were collected at baseline and weeks 12, 24, 48, 96, and 144.

### Definitions

The upper limit of normal (ULN) for AST and ALT was 40 IU/L. Virological response was defined as HBV DNA level of ≤ 20 IU/mL by real-time polymerase chain reaction assay [27, 28]. Biochemical response was defined as normalization of serum ALT level within ULN [29]. Significant decline in LS value at week 96 was defined as a 30% or more decrease in LS values from baseline [19, 20]. Previous studies have verified the accuracy of LS values in predicting the stage of histological liver fibrosis using the METAVIR scoring system [30–33]. Optimal cut-off points of LS values were 7.2 kPa for ≥ F2, 8.1 kPa for ≥ F3, and 11.1 kPa for ≥ F4 [31]. We defined mild fibrosis as F0–2, and significant fibrosis as F3–4. The fibrosis-4 (FIB-4) was determined using the proposed formula: FIB-4 = [(Age (years) × AST (IU/L)) / (platelet count (10^9^/L) × √ALT (IU/L))].

### Endpoints

The primary endpoint was the change in LS values at week 96 from baseline. The secondary endpoints were the changes in LS values at weeks 48 and 144 from baseline, and the predictors related to the decline in LS value at week 96 from baseline.

### Statistical analysis

The required number of study subjects was calculated based on a previous study analyzing changes in the LS values during antiviral therapy with lamivudine or entecavir [34]. We estimated that a sample size of 34 patients would provide 90% power to detect the decrease in LS value of 3.1 kPa at week 96 after initiating TDF therapy from baseline using a paired *t*-test with a two-sided significance level of 0.05.

Continuous variables are presented as the median with IQR and they were compared to baseline values using the repeated measures analysis of variance. Categorical variables are presented as frequencies and percentages and they were analyzed using the McNemar’s test. Among the variables that clinically affect liver fibrosis, Pearson correlation analysis was performed to explore covariables associated with the LS value reduction at week 96 from baseline. The independent and dependent variables of the Pearson correlation analysis were continuous variables following a normal distribution, and logarithmic transformation was performed for ALT and GGT variables that were not normally distributed based on the normality test. A stepwise multiple linear regression model was used to identify independent predictors associated with the decline in LS value at week 96 from baseline. *P* values < 0.05 were considered as statistically significant. Statistical analysis was conducted using the SPSS software (version 28.0; SPSS Inc., Chicago, Illinois, USA).

## Results

### Baseline characteristics

**Fig. 1:**
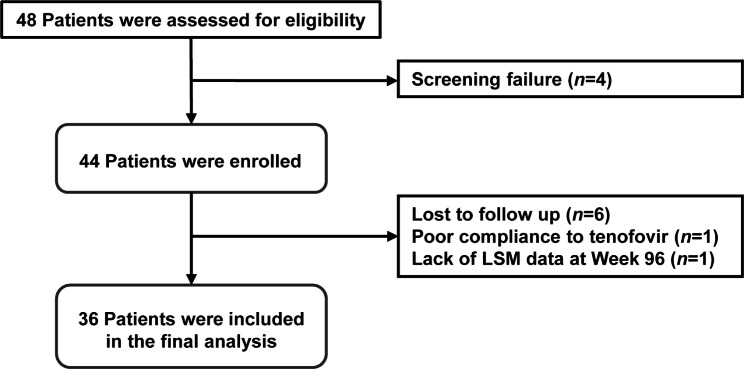
Flow diagram of study population. Abbreviations: LSM, liver stiffness measurement

A total of 48 treatment-naïve patients with CHB were assessed for eligibility, and 4 patients who did not meet the inclusion criteria at screening were excluded from the study. Among 44 patients who were enrolled and initiated TDF therapy, 8 patients were excluded due to loss to follow-up (n = 6), poor compliance to TDF (n = 1), and lack of LS value at week 96 (n = 1). Finally, 36 study patients completed 144 weeks of follow-up and were included in the final analysis (Fig. 1). TDF was administered orally in a daily dose of 300 mg for 144 weeks.

The baseline characteristics of 36 study patients are shown in Table 1. The median age was 46 (IQR, 34.5–55.8) years and 19 of 36 (52.8%) patients were men. Among the 36 study patients, 8 (22.2%) patients were clinically diagnosed with liver cirrhosis, and 19 (52.8%) patients were HBeAg-positive. The median serum levels of ALT and HBV DNA were 82 (IQR, 37.8–136.3) IU/L and 6.1 (IQR, 5.4–7.2) log_10_ IU/mL, respectively. The median LS value was 13.8 (IQR, 8.8–18.3) kPa.

**Table 1:** Baseline characteristics of the study population (n = 36)

Variable	Values
Age	46 (34.5–55.8)
Sex	
Male	19 (52.8)
Female	17 (47.2)
Body mass index (kg/m^2^)	24.5 (22.6–26.7)
Cirrhosis	8 (22.2)
Laboratory variables	
Platelet count (10^9^/L)	146 (107.8–185.5)
International normalized ratio	1.1 (1.1–1.2)
Aspartate aminotransferase (IU/L)	56 (42.3–117.3)
Alanine aminotransferase (IU/L)	82.5 (37.8–136.3)
Albumin (g/dL)	4.2 (3.9–4.6)
Fasting blood glucose (mg/dL)	96 (90–104)
GGT (IU/L)	45.5 (29.5–77)
HBV DNA (log_10_IU/ml)	6.1 (5.4–7.2)
HBeAg positivity (%)	19 (52.8)
Liver stiffness measurement	
Liver stiffness values (kPa)	13.8 (8.8–18.3)
Fibrosis stage	
Mild fibrosis	9 (25)
Significant fibrosis	27 (75)

NOTE: Variables are expressed as median (interquartile range) or n (%)

Abbreviations: GGT, gamma-glutamyl transferase; HBeAg, hepatitis B e antigen

### Changes in LS values and FIB-4 scores during follow-up

**Fig. 2:**
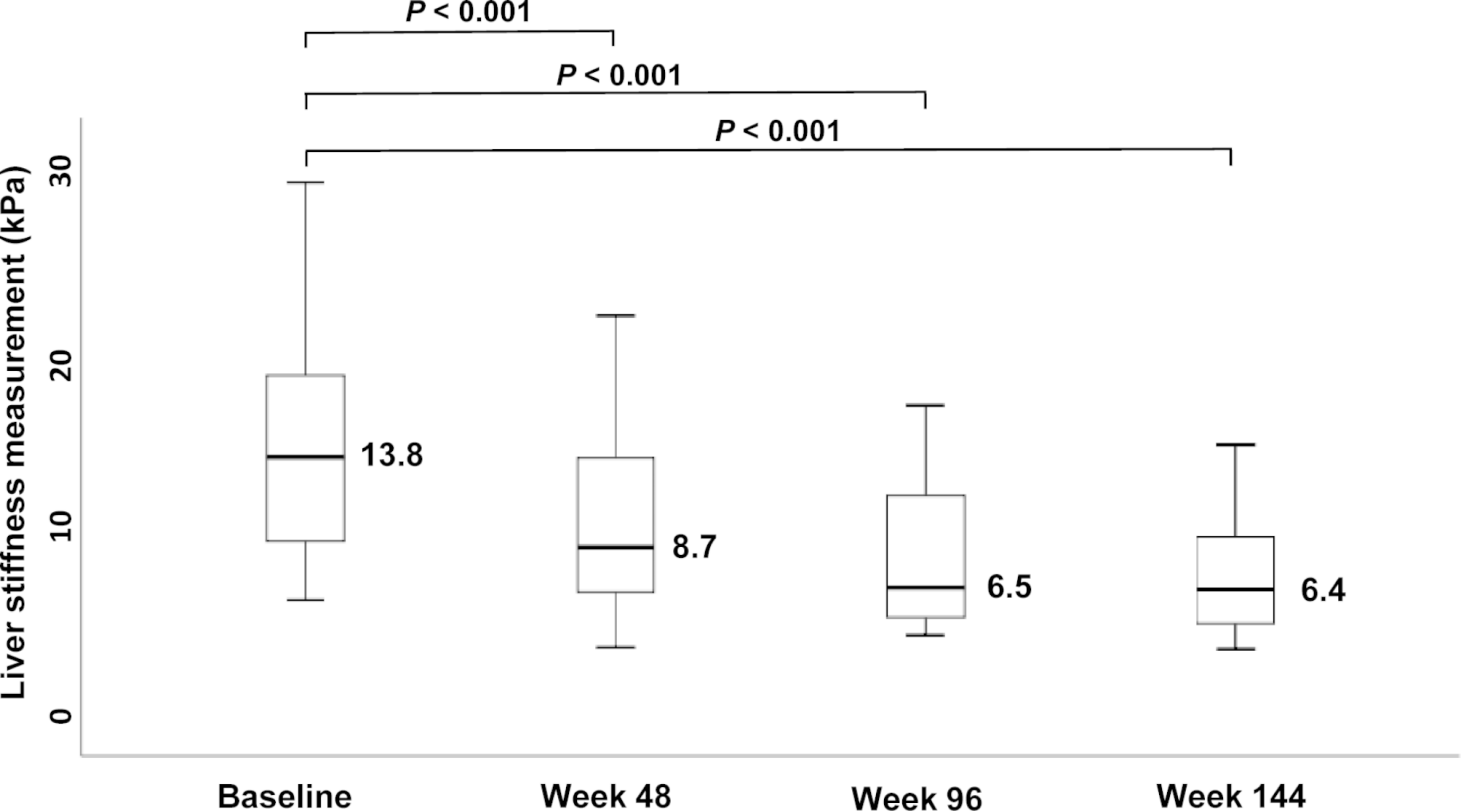
Box plots represented changes in LS values at baseline, week 48, week 96, and week 144 in the study population. *P* value measured is from a repeated measures analysis of variance. Abbreviations: LS, liver stiffness

Table 2 shows the comparison of LS values and FIB-4 scores at baseline and weeks 48, 96 and 144. During antiviral therapy with TDF, the median LS values decreased from 13.8 (IQR, 8.8–18.3) kPa at baseline to 8.7 (IQR, 6.2–13.8) kPa, 6.5 (IQR, 4.8–11.8) kPa, and 6.4 (IQR, 4.4–9.3) kPa at weeks 48, 96 and 144, respectively (all *P* < 0.001) in the overall study population (Fig. 2; Table 2). FIB-4 scores decreased during TDF therapy in the overall study population, and HBeAg-positive and -negative patients (Table 2). Additionally, decrease in LS values at week 96 was statistically significant observed among both HBeAg-positive and -negative patients (Table 2). Furthermore, the median LS value at week 96 declined compared to those at baseline among cirrhotic patients (from 19.7 kPa to 12.1 kPa; *P* = 0.07) and among non-cirrhotic patients (from 12.5 kPa to 6.2 kPa; *P* = 0.002). Additionally, patients were stratified according to the baseline ALT level, and the changes in LS value and FIB-4 scores at weeks 48, 96 and 144 after TDF therapy are shown in Supplementary Table S1. Among 26 patients who had elevated ALT level at baseline, the LS values at weeks 48, 96 and 144 decreased significantly from baseline (all *P* < 0.001; Supplementary Table S1). Among 10 patients who had normal ALT level at baseline, LS values decreased during TDF therapy, but the differences were not statistically significant. Supplementary Table S2 shows the trends of LS values and FIB-4 scores at 48, 96, and 144 weeks, divided into groups with and without biochemical responses at 96 weeks after TDF therapy. In the group that obtained biochemical response at 96 week, LS values significantly decreased over baseline and week 48, 96, and 144, but decrease of FIB-4 scores was not significant. In the group without biochemical response at 96 week, LS values and FIB-4 scores decreased but without statistical significance.

**Table 2:** Comparison of LS values and FIB-4 scores between baseline, week 48, week 96, and week 144 in the overall population and the HBeAg-positive and -negative patients

	Baseline	Week 48		Week 96		Week 144	
*Overall population (n = 36)*			P-value*		P-value*		P-value*
Liver stiffness measurement							
Liver stiffness values (kPa)	13.8 (8.8–18.3)	8.7 (6.2–13.8)	< 0.001	6.5 (4.8–11.8)	< 0.001	6.4 (4.4–9.3)	< 0.001
Fibrosis stage			0.008		< 0.001		< 0.001
Mild fibrosis	9 (25.0)	19 (52.8)		24 (66.7)		29 (80.6)	
Significant fibrosis	27 (75.0)	17 (47.2)		12 (33.3)		7 (19.4)	
FIB-4	2.3 (1.7–4.5)	1.4 (1.0–2.6)	0.06	1.1 (0.8–2.7)	0.03	1.2 (0.8–2.1)	0.01
***HBeAg-positive patients (n = 19)***							
Liver stiffness measurement							
Liver stiffness values (kPa)	14.4 (9.6–28.9)	10.5 (5.3–16.9)	< 0.001	7.7 (4.6–12.0)	< 0.001	5.7 (3.5–9.3)	< 0.001
Fibrosis stage			0.06		0.03		0.008
Mild fibrosis	4 (21.1)	9 (47.4)		12 (63.2)		15 (78.9)	
Significant fibrosis	15 (78.9)	10 (52.6)		7 (36.8)		4 (21.1)	
FIB-4	2.3 (1.6–4.6)	1.3 (1.0–3.8)	0.05	1.1 (0.7–3.4)	0.006	1.1 (0.8–3.0)	0.005
***HBeAg-negative patients (n = 17)***							
Liver stiffness measurement							
Liver stiffness values (kPa)	12.1 (7.1–17.2)	8.6 (6.4–12.3)	0.03	6.3 (5.3–11.7)	0.002	6.6 (4.9–9.0)	0.003
Fibrosis stage			0.25		0.03		0.008
Mild fibrosis	5 (29.4)	10 (58.8)		12 (70.6)		14 (82.4)	
Significant fibrosis	12 (70.6)	7 (41.2)		5 (29.4)		3 (17.6)	
FIB-4	2.3 (1.8–3.6)	1.5 (1.1–2.1)	0.68	1.1 (0.8–2.2)	0.50	1.4 (0.9–1.6)	0.39

NOTE: Variables are expressed as median (interquartile range) or n (%).

Abbreviations: FIB-4, Fibrosis-4; HBeAg, hepatitis B e antigen.

*Calculated compared to baseline value.

### Treatment response during TDF therapy

Virological response was achieved in 29 (80.6%), 34 (94.4%), and 35 (97.2%) patients at weeks 48, 96 and 144, respectively. Biochemical response was achieved in 20 (76.9%), 20 (76.9%), and 22 (84.6%) patients at weeks 48, 96, and 144, respectively. Among 19 HBeAg-positive patients, HBeAg seroclearance occurred in 4 (21.1%) and 7 (36.8%) patients at weeks 96 and 144, respectively. During the entire follow-up period of 144 weeks, resistance to the NAs or virological breakthrough was not observed in any study patient receiving antiviral therapy with TDF.

### Predictors associated with the decline in LS value at week 96 from baseline

At week 96, 21 of 36 (58.3%) patients showed a significant decline in LS value from baseline. Pearson correlation analysis revealed that the reduction in LS value at week 96 from baseline was positively correlated with the logarithmic transformed serum level of GGT at baseline (r value, 0.52; *P* = 0.001) and baseline LS value (r value, 0.94; *P* < 0.001) (Table 3). The statistically significant factors in the Pearson correlation analysis were included in the multiple linear regression model to identify predictive factors associated with the decline in LS value at week 96 from baseline. A higher baseline LS value was revealed to be a single independent predictor for the LS value reduction at week 96 from baseline (Table 4). R^2^ and adjusted R^2^ values indicating the explanatory power of this model were 0.89 and 0.88, respectively. According to the analysis, the multiple linear regression equation obtained was:

reduction in LS value = − 9.58 + 0.80 × baseline LS value.

**Table 3:** Correlation between the baseline characteristics and the reduction in LS values at week 96 from baseline

Variables			r^a^	P-value
Age			-0.11	0.51
Body mass index (kg/m^2^)			0.17	0.33
Platelet count (10^9^/L)			-0.09	0.61
Alanine aminotransferase (log_10_IU/L)			0.29	0.09
γ–glutamyl transferase (log_10_IU/L)			0.52	0.001
HBV DNA (log_10_IU/ml)			0.06	0.74
HBeAg positivity			0.33	0.05
Liver stiffness values (kPa)			0.94	< 0.001

^a^Pearson correlation coefficient

**Table 4:** Multiple linear regression analysis for predictors related to the differences in LS values at week 96 compared to baseline.

	Non-standardized coefficient		Standard coefficient			
Variables	*β* ^*a*^	SE	*β*	*t* ^b^	95% CI	P-value
Constant	-9.58	3.32		-2.89	-16.33–2.83	0.007
GGT (log_10_IU/L)	3.62	2.16	0.11	1.67	-0.78–8.02	0.10
Liver stiffness values (kPa)	0.80	0.06	0.89	13.50	-0.68–0.91	< 0.001

Abbreviations: GGT, gamma-glutamyl transferase; SE, standard error; CI, confidence interval.

^a^Estimated regression coefficient.

^b^Test statistic. The larger the test statistic, the less likely it is that the results occurred by change.

## Discussion

In this prospective observational study including 36 treatment-naïve CHB patients receiving long-term antiviral therapy with TDF, we demonstrated that the LS values decreased significantly over time during the follow-up period of 144 weeks and 58.3% of the study patients achieved significant decline in LS value at week 96. The significant decreases in LS value at week 96 were observed among both HBeAg-positive and -negative patients. A higher baseline LS value was determined to be an independent predictor for the reduction in LS value at week 96 from baseline.

Several previous studies have reported that the LS values decreased during long-term antiviral therapy [17, 19, 20]. A retrospective cohort study in Taiwan including 233 CHB patients who received entecavir therapy found that the LS values significantly declined among both non-cirrhotic and cirrhotic patients [17]. The improvement of LS value during 3-year entecavir therapy in CHB patients was confirmed in a prospective cohort study conducted in Korea [19]. Those previous studies showed that a higher initial LS value was associated with LS reduction during long-term antiviral therapy with entecavir. However, since TDF is another antiviral agent recommended by current international practice guidelines, it is necessary to revalidate whether liver fibrosis is reduced during long-term TDF therapy. We recently reported another prospective multicenter study on 131 patients with HBV-related cirrhosis who underwent TDF therapy [20]. In that previous study, we enrolled treatment-naïve CHB patients who were histologically diagnosed with cirrhosis through liver biopsy and followed up for 3 years during TDF therapy. After 3 years of antiviral treatment with TDF, the LS value significantly decreased (from 14.7 to 8.6 kPa; *P* < 0.001) and 96 of 131 (73.3%) patients achieved LS improvement, defined as LS value decline ≥ 30% from baseline [20]. It is worthy of investigation to clarify whether the LS value improves during TDF therapy in treatment-naive CHB patients, including non-cirrhosis patients; thus, we conducted the current prospective study. Our study findings are generally consistent with those of previous studies. In addition, we verified that virological and biochemical responses were achieved in most study patients, and no virological breakthrough occurred during the 144-week TDF therapy. Collectively, these findings suggest that LS improvement might be expected in addition to achieving virological and biochemical responses through long-term antiviral treatment with TDF in CHB patients, regardless of cirrhotic status.

Long-term antiviral treatment has been shown to reduce hepatic necroinflammation and regress liver fibrosis [10, 12, 13]. The association between antiviral therapy with lamivudine or adefovir and fibrosis regression was demonstrated, but the clinical benefit of long-term treatment with lamivudine or adefovir is inevitably limited due to their lower barrier to resistance [12, 13]. In a previous study on the efficacy of 3-year lamivudine treatment, a histological improvement was observed in 73% of patients with cirrhosis. However, lamivudine-resistant HBV variants were found in 67% of patients treated with lamivudine, resulting in a loss of histological benefit from long-term antiviral therapy [13]. Similarly, although a histological regression of liver fibrosis was found in 60% of patients receiving 5-year adefovir treatment, genotypic resistance to adefovir occurred in 20% of patients, negating the clinical benefit of histological improvement [12]. In contrast, tenofovir resistance has been reported to be extremely rare [7, 10, 12, 35]. A higher antiviral potency and a high genetic barrier to resistance of TDF can make long-term therapy feasible, leading to attenuation of hepatic necroinflammation and regression of fibrosis [8, 10].

A higher LS value at baseline was the sole predictor associated with the decline in LS values at week 96 from baseline in our current study. This finding reaffirmed the results from the aforementioned previous study identifying the baseline LS value as the only predictive factor for significant decline in LS value during NAs therapy; however, this finding needs to be interpreted carefully. It has been reported that the LS value tends to be overestimated compared to the actual liver fibrosis in CHB patients in immune-active phase, because liver elasticity decreases and LS increases with severe hepatic inflammation [17, 20, 36, 37]. However, although we enrolled treatment-naïve CHB patients that were indicated for antiviral therapy without limiting ALT values at baseline, multiple linear regression analysis demonstrated that the baseline ALT value was not significantly associated with the LS value reduction. Based on this finding, it might be inferred that the baseline ALT levels did not significantly affect LS values at baseline in our present study. This is presumably due to the small number of the study population.

Although our present study had the strengths of a prospective observational design and long-term follow-up period, it had several limitations. First, since the sample size of our present study was calculated based on the aim of verifying the LS reduction after a 96-week TDF therapy, it may not be sufficient to identify predictors associated with a significant decline in LS value. Therefore, future large-scale studies are warranted to validate our results. Second, changes were measured with LS values as an indirect indicator of liver fibrosis, but validation was not performed with other histologic biomarkers except FIB-4. The pathophysiology of liver fibrogenesis has dynamic kinetics and it is an elaborate and collective process by cells regulating the accumulation of molecular, cellular, and extracellular matrix components; activation of hepatic myofibroblasts; inflammation; and angiogenesis. Therefore, the combination of non-invasive tools for evaluating complex processes of liver fibrosis, such as direct and indirect serum biomarkers, and other imaging techniques could be considered available in the study of small-sized samples.

## Conclusion

In this prospective cohort of treatment-naïve CHB patients, the LS values decreased significantly over time during the 144-week TDF therapy. Our study results provided additional evidence that supports the effectiveness of long-term TDF therapy in reducing liver fibrosis.

## Electronic supplementary material

Below is the link to the electronic supplementary material.

Supplementary Material 1

## Data Availability

The corresponding author (Yun Bin Lee) had full access to all the data in the study and takes responsibility for the integrity of the data and the accuracy of the data analysis.

## Abbreviations

ALT: Alanine aminotransaminase
AST: Aspartate aminotransferase
BMI: Body mass index
CHB: Chronic hepatitis B
FIB-4: Fibrosis-4
HBeAg: Hepatitis B e antigen
HBsAg: Hepatitis B surface antigen
HBV: Hepatitis B virus
HCC: Hepatocellular carcinoma
IQR: Interquartile range
LS: Liver stiffness
LSM: Liver stiffness measurement
NAs: Nucleos(t)ide analogues
TDF: Tenofovir disoproxil fumarate
TE: Transient elastography
ULN: Upper limit of normal
