# Supplementary material for: Changes in liver stiffness values assessed using transient elastography in chronic hepatitis B patients treated with tenofovir disoproxil fumarate: a prospective observational study
Source: BMC Gastroenterol. 2023 Jun 15;23:210. doi: 10.1186/s12876-023-02846-9 (PMC10273722; doi:10.1186/s12876-023-02846-9)
Supplement: Supplementary file 1 — Supplementary Material 1 [file 12876_2023_2846_MOESM1_ESM.docx]

**Online Supplementary Material**

Heejin Cho, et al. Changes in liver stiffness values assessed using transient elastography in chronic hepatitis B patients treated with tenofovir disoproxil fumarate: a prospective observational study

**Supporting Table 1.** Comparisons of LS values and FIB-4 scores between baseline, week 48, week 96, and week 144 in the strata according to the baseline ALT level.

**Supporting Table 2.** Comparison of LS values and FIB-4 scores between baseline, week 48, week 96, and week 144 in the strata according to biochemical response at week 96 after TDF therapy.

**Revised Supplementary Table S1**. Comparisons of LS values and FIB-4 scores between baseline, week 48, week 96, and week 144 in the strata according to the baseline ALT level.

|  |  |  |  | **Baseline** | **Week 48** | | **Week 96** | | **Week 144** | |
| --- | --- | --- | --- | --- | --- | --- | --- | --- | --- | --- |
| ***Patients with elevated ALT at baseline (n=26)*** | | | |  |  | **P-value*** |  | **P-value*** |  | **P-value*** |
| Liver stiffness measurement | | |  |  |  |  |  |  |  |  |
|  | Liver stiffness values (kPa) | | | 12.9 (8.1–21.4) | 7.7 (5.2–12.2) | <0.001 | 6.2 (4.7–9.0) | <0.001 | 5.1 (3.9–8.4) | <0.001 |
|  | Fibrosis stage | |  |  |  | 0.02 |  | 0.002 |  | <0.001 |
|  |  | Mild fibrosis | | 6 (23.1) | 15 (57.7) |  | 20 (76.9) |  | 22 (84.6) |  |
|  |  | Significant fibrosis | | 20 (76.9) | 11 (42.3) |  | 6 (23.1) |  | 4 (15.4) |  |
| FIB-4 |  |  | | 2.0 (1.5–4.2) | 1.2 (0.9–1.6) | 0.14 | 1.0 (0.7–1.6) | 0.08 | 1.0 (0.7–1.5) | 0.04 |
| ***Patients with normal ALT at baseline (n=10)*** | | | |  |  |  |  |  |  |  |
| Liver stiffness measurement | | |  |  |  |  |  |  |  |  |
|  | Liver stiffness values (kPa) | | | 14.9 (10.6–17.8) | 13.9 (7.6–18.1) | 1.00 | 11.0 (6.3–14.1) | 0.71 | 9.0 (6.5–13.4) | 0.68 |
|  | Fibrosis stage | |  |  |  | 1.00 |  | 0.50 |  | 0.25 |
|  |  | Mild fibrosis | | 2 (20.0) | 4 (40.0) |  | 4 (40.0) |  | 7 (70.0) |  |
|  |  | Significant fibrosis | | 8 (80.0) | 6 (60.0) |  | 6 (60.0) |  | 3 (30.0) |  |
| FIB-4 |  |  | | 3.0 (2.0–5.3) | 2.7 (1.2–4.2) | 1.00 | 2.7 (1.1–4.7) | 0.35 | 2.7 (1.1–4.7) | 0.15 |

NOTE: Variables are expressed as median (interquartile range) or n (%).

Abbreviations: ALT, Alanine transferase; FIB-4, Fibrosis-4

*Calculated compared to baseline value.

**Revised Supplementary Table S2**. Comparisons of LS values and FIB-4 scores between baseline, week 48, week 96, and week 144 in the strata according to biochemical response at week 96 after TDF therapy.

|  |  |  |  | **Baseline** | **Week 48** | | **Week 96** | | **Week 144** | |
| --- | --- | --- | --- | --- | --- | --- | --- | --- | --- | --- |
| ***Patients with biochemical response (n=20)*** | | | |  |  | **P-value*** |  | **P-value*** |  | **P-value*** |
| Liver stiffness measurement | | |  |  |  |  |  |  |  |  |
|  | Liver stiffness values (kPa) | | | 12.4 (7.3–17.7) | 6.6 (5.0–10.8) | <0.001 | 5.3 (4.6–8.7) | <0.001 | 4.9 (3.8–8.0) | <0.001 |
|  | Fibrosis stage | |  |  |  | 0.03 |  | 0.008 |  | 0.002 |
|  |  | Mild fibrosis | | 6 (30.0) | 12 (60.0) |  | 16 (70.0) |  | 17 (85.0) |  |
|  |  | Significant fibrosis | | 14 (70.0) | 8 (40.0) |  | 4 (30.0) |  | 3 (15.0) |  |
| FIB-4 |  |  | | 1.9 (1.4–3.7) | 1.1 (0.7–1.6) | 0.25 | 1.0 (0.5–1.5) | 0.18 | 1.0 (0.6–1.3) | 0.12 |
| ***Patients without biochemical response (n=6)*** | | | |  |  |  |  |  |  |  |
| Liver stiffness measurement | | |  |  |  |  |  |  |  |  |
|  | Liver stiffness values (kPa) | | | 17.7 (10.6–28.2) | 10.4 (6.5–14.9) | 0.67 | 7.0 (5.8–12.5) | 0.52 | 6.8 (4.0–9.7) | 0.49 |
|  | Fibrosis stage | |  |  |  | 1.00 |  | 0.50 |  | 0.25 |
|  |  | Mild fibrosis | | 1 (17.0) | 3 (50.0) |  | 4 (66.7) |  | 5 (83.0) |  |
|  |  | Significant fibrosis | | 5 (83.0) | 3 (50.0) |  | 2 (33.3) |  | 1 (17.0) |  |
| FIB-4 |  |  | | 2.7 (2.0–4.8) | 1.5 (1.2–3.3) | 0.99 | 1.0 (0.9–3.4) | 0.83 | 1.4 (0.8–2.2) | 0.25 |

NOTE: Variables are expressed as median (interquartile range) or n (%).

Abbreviations: FIB-4, Fibrosis-4; TDF, Tenofovir disoproxil fumarate

*Calculated compared to baseline value.
